# A predictive nomogram for mortality of cancer patients with invasive candidiasis: a 10-year study in a cancer center of North China

**DOI:** 10.1186/s12879-021-05780-x

**Published:** 2021-01-15

**Authors:** Ding Li, Tianjiao Li, Changsen Bai, Qing Zhang, Zheng Li, Xichuan Li

**Affiliations:** 1 Department of Clinical Laboratory, Tianjin Medical University Cancer Institute and Hospital, National Clinical Research Center for Cancer, Key Laboratory of Cancer Prevention and Therapy, Tianjin’s Clinical Research Center for Cancer, Huanhu West Road, Hexi District, Tianjin, 300060 China; 2 State Key Laboratory of Medicinal Chemical Biology, College of Pharmacy, Nankai University, Tianjin, China; 3 Tianjin Key Laboratory of Animal and Plant Resistance, College of Life Sciences, Tianjin Normal University, Binshuixi Road, Tianjin, 300387 Xiqing District China

**Keywords:** Invasive candidiasis, Mortality, Predictive nomogram, *Candida*, 30-day death

## Abstract

**Background:**

Invasive candidiasis is the most common fungal disease among hospitalized patients and continues to be a major cause of mortality. Risk factors for mortality have been studied previously but rarely developed into a predictive nomogram, especially for cancer patients. We constructed a nomogram for mortality prediction based on a retrospective review of 10 years of data for cancer patients with invasive candidiasis.

**Methods:**

Clinical data for cancer patients with invasive candidiasis during the period of 2010–2019 were studied; the cases were randomly divided into training and validation cohorts. Variables in the training cohort were subjected to a predictive nomogram based on multivariate logistic regression analysis and a stepwise algorithm. We assessed the performance of the nomogram through the area under the receiver operating characteristic (ROC) curve (AUC) and decision curve analysis (DCA) in both the training and validation cohorts.

**Results:**

A total of 207 cases of invasive candidiasis were examined, and the crude 30-day mortality was 28.0%. *Candida albicans* (48.3%) was the predominant species responsible for infection, followed by the *Candida glabrata* complex (24.2%) and *Candida tropicalis* (10.1%). The training and validation cohorts contained 147 and 60 cases, respectively. The predictive nomogram consisted of bloodstream infections, intensive care unit (ICU) admitted > 3 days, no prior surgery, metastasis and no source control. The AUCs of the training and validation cohorts were 0.895 (95% confidence interval [CI], 0.846–0.945) and 0.862 (95% CI, 0.770–0.955), respectively. The net benefit of the model performed better than “treatment for all” in DCA and was also better for opting low-risk patients out of treatment than “treatment for none” in opt-out DCA.

**Conclusion:**

Cancer patients with invasive candidiasis exhibit high crude mortality. The predictive nomogram established in this study can provide a probability of mortality for a given patient, which will be beneficial for therapeutic strategies and outcome improvement.

## Background

Invasive candidiasis refers to bloodstream (that is, candidemia) and deep-seated infections by *Candida spp.*, a common opportunistic pathogenic fungus that is commensal on the human body surface, including the skin, oral cavity, intestines and vagina [1]. *Candida* ranks as the fourth most prevalent nosocomial pathogen of bloodstream infection in the United States and seventh to tenth in population-based studies, with the *C. albicans*, *C. tropicalis*, *C. glabrata* and *C. parapsilosis* complex comprising the vast majority causal agents of the disease [2, 3]. Although the diagnostic tools and management strategies of invasive candidiasis have been improved, it is still a deadly infection found all over the world, and the mortality can reach 40% and even higher among patients with malignancy or those who are critically ill [4, 5].

Invasive candidiasis represents a major challenge among healthcare-related infections due to its difficult diagnostic and therapeutic management [6]. Therefore, a better understanding of the underlying risk factors for the development of infection and mortality is of high clinical importance. A number of studies have found that mortality independently increases with elderly age, renal failure, malignant diseases, central venous catheterization (CVC), steroid therapy, admission to an intensive care unit (ICU), use of total parenteral nutrition (TPN), low lymphocyte count, gastrointestinal source of candidemia, or previous exposure to antibiotics [1, 7, 8]. However, these predictors contribute little to obtaining a better prognosis and always vary among populations. Thus, we speculated that a model combining different risk factors (cumulative number) might provide a better prediction for the outcome of invasive candidiasis than a single factor.

A large proportion of cancer patients with both hematologic and solid malignancies are susceptible to invasive candidiasis and have poor outcomes [9]. In other words, invasive candidiasis greatly reduces the survival rate of cancer patients. To our knowledge, research on prognosis prediction for invasive candidiasis among cancer patients is limited, and predictive models are rarely studied. In the present study, we constructed a predictive nomogram based on prognosis predictors of cancer patients with invasive candidiasis and showed that this nomogram has a good mortality prediction ability in both training and validation cohorts. Thus, we believe that the nomogram constructed in the study will provide a better prognosis prediction than a single predictor and will benefit treatment strategies (such as strengthening source control and intravenous therapy) and increase the survival rate among cancer patients with invasive candidiasis.

## Methods

### Study design

This was a retrospective observational study, and data were collected from Tianjin Medical University Cancer Institute and Hospital, among the top cancer institutes in China. To construct the predictive nomogram for 30-day death of invasive candidiasis among cancer patients, we first randomly divided patient data into training and validation cohorts. The predictive nomogram model was constructed according to the data in the training cohort, and the data in the validation cohort were verified by using the same regression equations that were constructed for the training cohort. The ability of this predictive nomogram was assessed by using a calibration plot of the area under the receiver operating characteristic (ROC) curve (AUC), and the clinical usefulness was examined via net benefit by using decision curve analysis (DCA).

This study obtained permission from the Bioethics Committee of Tianjin Medical University Cancer Institute and Hospital and participants (consent to participate was obtained from all participants or their parents or legal guardians for participants under 16 years old) to review patient records and use the data.

### Data collection

**Fig. 1:**
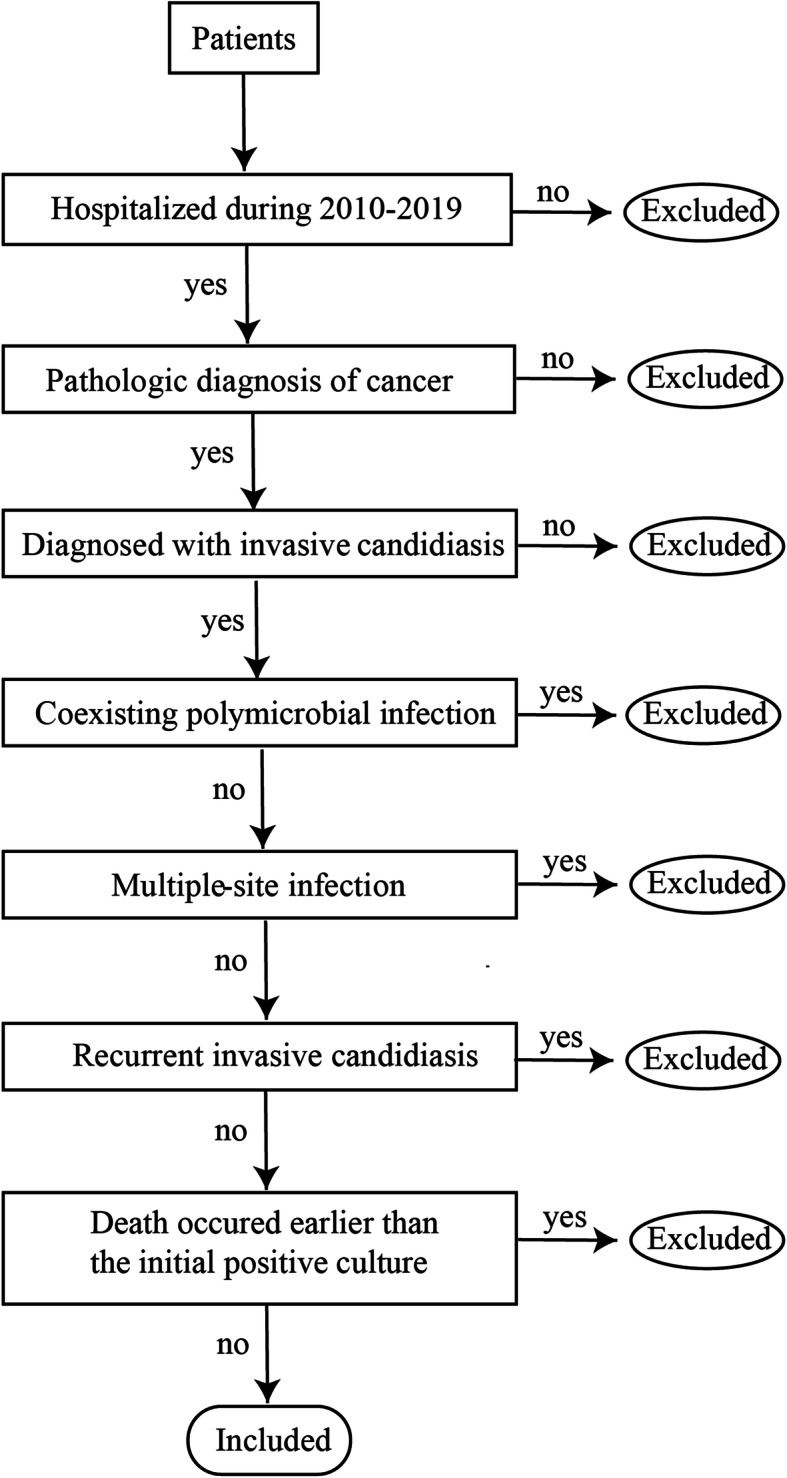
Flow chart of patients excluded due to each criterion

Cancer patients who were hospitalized during the period from 2010 to 2019 and had been diagnosed with invasive candidiasis were eligible for inclusion. Patients without a pathologic diagnosis of cancer, with polymicrobial or multiple-site infections, who had recurrent invasive candidiasis (occurring more than 30 days after the initial episode), or who died earlier than the initial positive culture were excluded; a flow chart of patients excluded for each criterion is shown in Fig. 1. *Candida* species were evaluated. Clinical data collected from medical reports included age, gender, length of hospital stay (LOS) to the initial positive culture, types of cancer, site of infection, tumor metastasis, predisposing factors (chemotherapy, neutropenia, surgery, prior antibiotic use, CVC retention > 7 days, urinary catheter retention > 2 days, nasogastric tube retention > 3 days, ICU admitted > 3 days, TPN administration > 5 days) present at or within the last 30 days prior to the initial positive culture, comorbidities (diabetes mellitus, live dysfunction, kidney dysfunction, respiratory dysfunction, cardiovascular dysfunction) present within the last 30 days prior to the initial positive culture or during the progression of the invasive candidiasis, and antifungal treatment (fluconazole prophylaxis before confirmation of invasive candidiasis, timely antifungal treatment and source control after diagnosis). All data and outcomes were collected on day 30 after the date of the initial positive culture or death if this occurred earlier. There were no missing values in this study.

### Definitions

A case diagnosed as invasive candidiasis was defined based on isolation of *Candida spp.* from bloodstream or other normally sterile sites, and isolation of *Candida spp.* from bloodstream was also called bloodstream infection [5, 10]. Polymicrobial infection referred to infection by more than one pathogen at the same time. Site of infection referred to the site from which *Candida* was isolated, including the bloodstream, intra-abdominal, intrathoracic and intrapelvic cavities. All deep-seated samples were obtained via paracentesis or surgery. Types of cancer were differentially diagnosed by pathological examination. Cancers occurring in the stomach, duodenum, colon or rectum were referred to as gastrointestinal cancer; cancers occurring in the bile duct, liver or pancreas were referred to as hepatic-pancreatic cancer. Neutropenia was defined as an absolute neutrophil count of < 1.5 × 10^9^/L. Comorbidities were determined by the attending physician as follows: 1) insulin dependent diabetes; 2) decompensated cirrhosis; 3) episodes of hepatic failure; 4) hepatic encephalopathy; 5) dialysis dependent renal disease; 6) respiratory dysfunction including dyspnea on exertion, chronic respiratory hypoxia, or pulmonary hypertension; 7) cardiovascular dysfunction was defined as New York Heart Association grades III to IV [11, 12]. Crude mortality referred to the rate of death within 30 days after the initial positive culture. Predictors were specified with worse outcomes in the study.

### *Candida spp.* identification

Blood samples (8–10 ml) were collected and autocultured using a BACTEC 9050, 9120 or FX (Becton–Dickinson, Franklin Lakes, NJ, USA) for 5 days; positive samples were subcultured on blood agar (JinZhangKeJi, Tianjin, China) at 35 °C for 24–48 h. Other sterile sourced samples were inoculated on agar with 5% sheep blood (JinZhangKeJi, Tianjin, China) for 24–48 h at 35 °*C. candida spp.* were preliminarily identified by Gram staining and then isolated using Sabourauds agar (JinZhangKeJi, Tianjin, China). Species identification was performed with a VITEK-2 Compact or VITEK-2 (bioMérieux AS, Marcy I’Etoile, France) by using purified yeast colonies.

### Statistical analysis

Univariate analysis of categorical variables was performed using Fisher’s exact test with SPSS 20.0 software (SPSS Inc., Chicago, IL, USA), and all tests were 2-tailed. For dividing the data randomly, multivariable logistic regression, stepwise algorithm, nomogram construct, DCA, calibration and ROC analysis were performed with R version 4.0.2 or 3.6.1 (R Core Team, Vienna, Austria). The significance level was set at *p* <  0.05.

To screen predictors in the training cohort, Fisher’s exact test was first performed. Then, clinical variables with *p* <  0.2 were used to construct a predictive model by multivariable logistic regression analysis, and the best model was selected via a stepwise algorithm based on the minimal Akaike’s information criterion (AIC). The variables in the predictive model we chose were applied to plot the predictive nomogram. The nomogram performance was composed of calibration and discrimination. Calibration was computed by comparing the predicted probability of 30-day death versus the actual probability of 30-day death in all patients, again using 1000 bootstrap resamples to reduce overfit bias, which would overstate the accuracy of the nomogram. Discrimination was quantified with the concordance index (C-index). We evaluated the predictive ability of the nomogram via ROC analysis in the training cohort and verified it in the validation cohort. For clinical usefulness, net benefit was examined via DCA individually in the training and validation cohorts.

## Results

### Distribution of *Candida spp.*

In total, 207 cases of invasive candidiasis were examined in the current investigation, and 207 strains of *Candida* were isolated. *C. albicans* was the predominant species (*n* = 100, 48.3%), followed by the *C. glabrata* complex (*n* = 50, 24.2%), *C. tropicalis* (*n* = 21, 10.1%), the *C. parapsilosis* complex (*n* = 15, 7.2%), *C. lusitaniae* (*n* = 9, 4.3%), *C. ciferrii* (n = 5, 2.9%), *C. famata* (n = 5, 2.4%) and *C. krusei* (n = 2, 0.1%).

### Clinical characteristics of patients and construction of a predictive nomogram

For the 207 cases, the median age was 63 years old (range from 1 to 88 years old), and the median LOS was 23 days (range from 3 to 105 days). A total of 58 patients died within 1 mon during the study period, leading to a crude mortality of 28.0%. The 207 cases were randomly split into two cohorts: a training cohort including 147 cases with 28.6% crude mortality and a validation cohort including 60 cases with 26.7% crude mortality. For the training cohort, the median age was 63 years old (range from 2 to 88 years old), and the median LOS was 24 days (range from 6 to 76 days). For the validation cohort, the median age was 61 years old (range from 1 to 86 years old), and the median LOS was 22 days (range from 3 to 105 days). None of the patients experienced organ infections due to *Candida spp.*, and none of patients with bloodstream infections from deep seated infections during the study period. The other detailed clinical characteristics and results of univariate analysis are shown in Table 1.

**Table 1:** Clinical characteristics of patients with invasive candidiasis for crude 30-day mortality in the training and validation cohorts

Clinical characteristics	Training cohort			Validation cohort		
	No. patients (*n* = 147)	30-day mortality (*n* = 42)	*p* value ^a^	No. patients (*n* = 60)	30-day mortality (*n* = 16)	*p* value ^a^
Male (%)	75 (51.0)	20 (47.6)	0.715	36 (60.0)	9 (56.3)	0.771
Age > = 65 years old (%)	68 (46.3)	21 (50.0)	0.587	22 (36.7)	6 (37.5)	1.000
LOS > = 30 days (%)	50 (34.0)	18 (42.9)	0.179	19 (31.7)	5 (31.3)	1.000
CVC > 7 days (%)	79 (53.7)	24 (57.1)	0.715	35 (58.3)	12 (75.0)	0.146
TPN > 5 days (%)	54 (36.7)	16 (38.1)	0.851	21 (35.0)	7 (43.8)	0.541
Urinary catheter > 2 days (%)	41 (27.9)	13 (31.0)	0.685	21 (35.0)	8 (50.0)	0.220
Nasogastric tube > 3 days (%)	60 (40.8)	14 (33.3)	0.270	24 (40.0)	7 (43.8)	0.771
No prior surgery (%)	68 (46.3)	30 (71.4)	< 0.001	41 (68.3)	14 (87.5)	0.066
Neutropenia (%)	9 (6.1)	3 (7.1)	0.715	3 (5.0)	2 (12.5)	0.171
Chemotherapy (%)	50 (34.0)	15 (35.7)	0.848	11 (18.3)	4 (25.0)	0.462
Metastasis (%)	50 (34.0)	28 (66.7)	< 0.001	24 (40.0)	9 (56.3)	0.145
ICU admitted > 3 days (%)	54 (36.7)	26 (61.9)	< 0.001	26 (43.3)	11 (68.8)	0.021
Previous antibiotics exposure (%)	104 (70.7)	31 (73.8)	0.691	41 (68.3)	10 (62.5)	0.550
Timely anti-fungal treatment	101 (68.7)	31 (73.8)	0.437	42 (70.0)	14 (87.5)	0.112
Fluconazole prophylaxis	11 (7.5)	5 (11.9)	0.295	9 (15.0)	5 (31.3)	0.048
No source control	59 (40.1)	32 (76.2)	< 0.001	29 (48.3)	11 (68.8)	0.081
Site of infection						
Bloodstream	51 (34.7)	25 (59.5)	< 0.001	22 (36.7)	11 (68.8)	0.005
Abdominal cavity	67 (45.6)	12 (28.6)	0.010	33 (55.0)	4 (25.0)	0.008
Thoracic cavity	17 (11.6)	3 (7.1)	0.397	2 (3.3)	0	1.000
Pelvic cavity	12 (8.2)	2 (4.8)	0.510	3 (5.0)	1 (6.3)	1.000
*Candida* species						
*Candida albicans*	72 (49.0)	18 (42.9)	0.367	28 (46.7)	4 (25.0)	0.077
*Candida glabrata* complex	40 (27.2)	16 (38.1)	0.068	10 (16.7)	6 (37.5)	0.017
*Candida tropicalis*	11 (7.5)	3 (7.1)	1.000	10 (16.7)	3 (18.8)	1.000
*Candida parapsilosis* complex	9 (6.1)	3 (7.1)	0.715	6 (10.0)	2 (12.5)	0.653
Other *Candida species* ^b^	15 (10.2)	2 (4.8)	0.233	6 (10.0)	1 (6.3)	1.000
Tumor types						
Gastrointestinal	50 (34.0)	13 (31.0)	0.702	23 (38.3)	7 (43.8)	0.765
Hepatic-pancreatic	57 (38.8)	19 (45.2)	0.351	22 (36.7)	4 (25.0)	0.367
Hematologic malignancy	9 (6.1)	4 (9.5)	0.276	4 (6.7)	2 (12.5)	0.287
Others ^c^	31 (21.1)	6 (14.3)	0.265	11 (18.3)	3 (18.8)	1.000
Co-morbidities						
Diabetes mellitus	28 (19.0)	9 (21.4)	0.647	14 (23.3)	4 (25.0)	1.000
Live dysfunction	30 (20.4)	9 (21.4)	0.825	14 (23.3)	4 (25.0)	1.000
Kidney dysfunction	23 (15.6)	8 (19.0)	0.462	4 (6.7)	3 (18.8)	0.054
Respiratory dysfunction	22 (15.0)	4 (9.5)	0.311	6 (10.0)	3 (18.8)	0.328
Cardiovascular dysfunction	20 (13.6)	8 (19.0)	0.286	1 (1.7)	1 (6.3)	0.267

*LOS* length of hospital stay, *CVC* central venous catheters, *TPN* total parenteral nutrition, *ICU* intensive care unit

^a^ Fisher exact test were compared between patient alive vs deceased in 30-day

^b^ In the training cohort: *Candida lusitaniae* (*n* = 7), *Candida ciferrii* (*n* = 3), *Candida famata* (*n* = 4) and *Candida krusei* (*n* = 1); In the validation cohort: *Candida lusitaniae* (*n* = 2), *Candida ciferrii* (*n* = 2), *Candida famata* (*n* = 1) and *Candida krusei* (*n* = 1)

^c^ In the training cohort: lung cancer (*n* = 11), ovarian cancer (*n* = 7), renal cancer (*n* = 3), thymoma (n = 3), breast cancer (*n* = 2), giiomas (*n* = 1), rhabdomyosarcoma (*n* = 1), endometrial cancer (*n* = 1), liposarcoma (*n* = 1), nephroblastoma (n = 1); In the validation cohort: lung cancer (*n* = 2), ovarian cancer (*n* = 2), renal cancer (*n* = 2), cervical cancer (*n* = 2), nephroblastoma (*n* = 1), liposarcoma (*n* = 1), fibrosarcoma (*n* = 1)

**Fig. 2:**
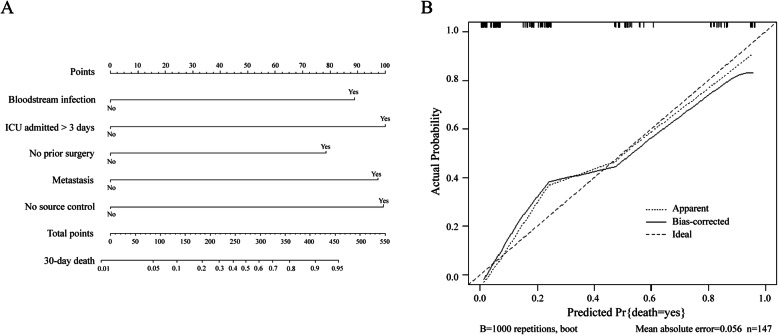
Predictive nomogram and calibration plot. **a**. A predictive nomogram for predicting 30-day death of invasive candidiasis among cancer patients. To estimate the probability of 30-day death for a given patient, the following were carried out: patient values at each axis were marked, a straight line perpendicular to the point axis was drawn, and the points for all variables were summed. Then, we summed the total points and drew a vertical line from the “total points” row to obtain the probability of 30-day death. **b**. A calibration plot of the predicted and observed probabilities of 30-day death of invasive candidiasis among cancer patients. The x-axis shows the predicted probability of 30-day death, and the y-axis shows the observed probability of 30-day death. The nomogram had a C-index of 0.895 and was well calibrated

To select the predictive model and plot the nomogram, we chose values of LOS > = 30 days, no prior surgery, metastasis, ICU admitted > 3 days, no source control, bloodstream infection, abdominal cavity infection, and *C. glabrata* complex as candidate predictors of which *p* <  0.2 in univariate analysis of the training cohort into multiple logistic regression and a stepwise algorithm. Then, we selected a predictive model containing predictors of bloodstream infection, ICU admitted > 3 days, no prior surgery, metastasis and no source control based on a minimal AIC (Table 2), and a nomogram using these predictors as weights was built (Fig. 2).

**Table 2:** Predictive model constructed by AIC in a Stepwise Algorithm in the training cohort

	Variables	OR	95% CI	*p* value ^a^	AIC
Model	Bloodstream infection	4.097	1.478–12.131	0.008	118.2
	ICU admitted > 3 days	4.877	1.812–14.257	0.002	
	No prior surgery	3.466	1.316–9.777	0.014	
	Metastasis	4.685	1.787–12.942	0.002	
	No source control	4.826	1.830–13.528	0.002	

^a^ Stepwise Algorithm, direction = “backward”

*OR* odds ratio, *CI* confidence interval, *AIC* Akaike’s Information Criterion

### ROC analysis

**Fig. 3:**
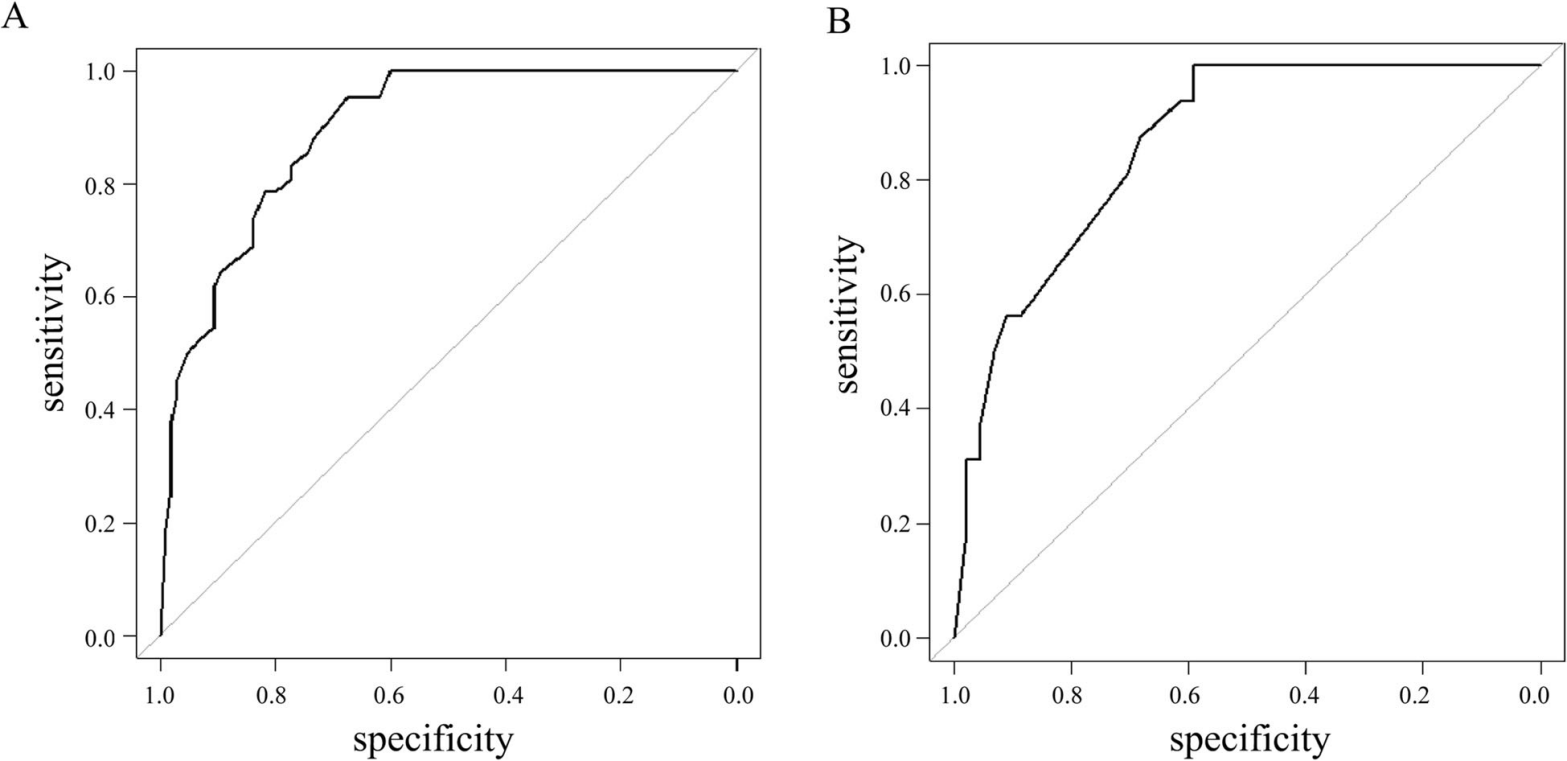
ROC curve for the predictive nomogram of 30-day death among cancer patients with invasive candidiasis. **a**. ROC curve for the training cohort; the AUC was 0.895 (95% confidence interval, 0.846–0.945). **b**. ROC curve for the validation cohort; the AUC was 0.862 (95% confidence interval, 0.770–0.955). Predictor variables are bloodstream infection, ICU admitted > 3 days, no prior surgery, metastasis and no source control

The predictive nomogram generates an individual numerical probability of 30-day death among cancer patients with invasive candidiasis, and the performance of the nomogram was evaluated by ROC analysis. Figure 3 shows the ROC curves for the training and validation cohorts. The AUCs of the training and validation cohorts were 0.895 (95% confidence interval [CI], 0.846–0.945) and 0.862 (95% CI, 0.770–0.955), respectively. In the training cohort, the cutoff value of the predictive score at the optimum point was 18.2, the specificity was 67.7%, and the sensitivity was 95.2%. For the validation cohort, the specificity was 61.4%, and the sensitivity was 93.8% when the cutoff value was set at 18.2.

### Decision curve analysis

**Fig. 4:**
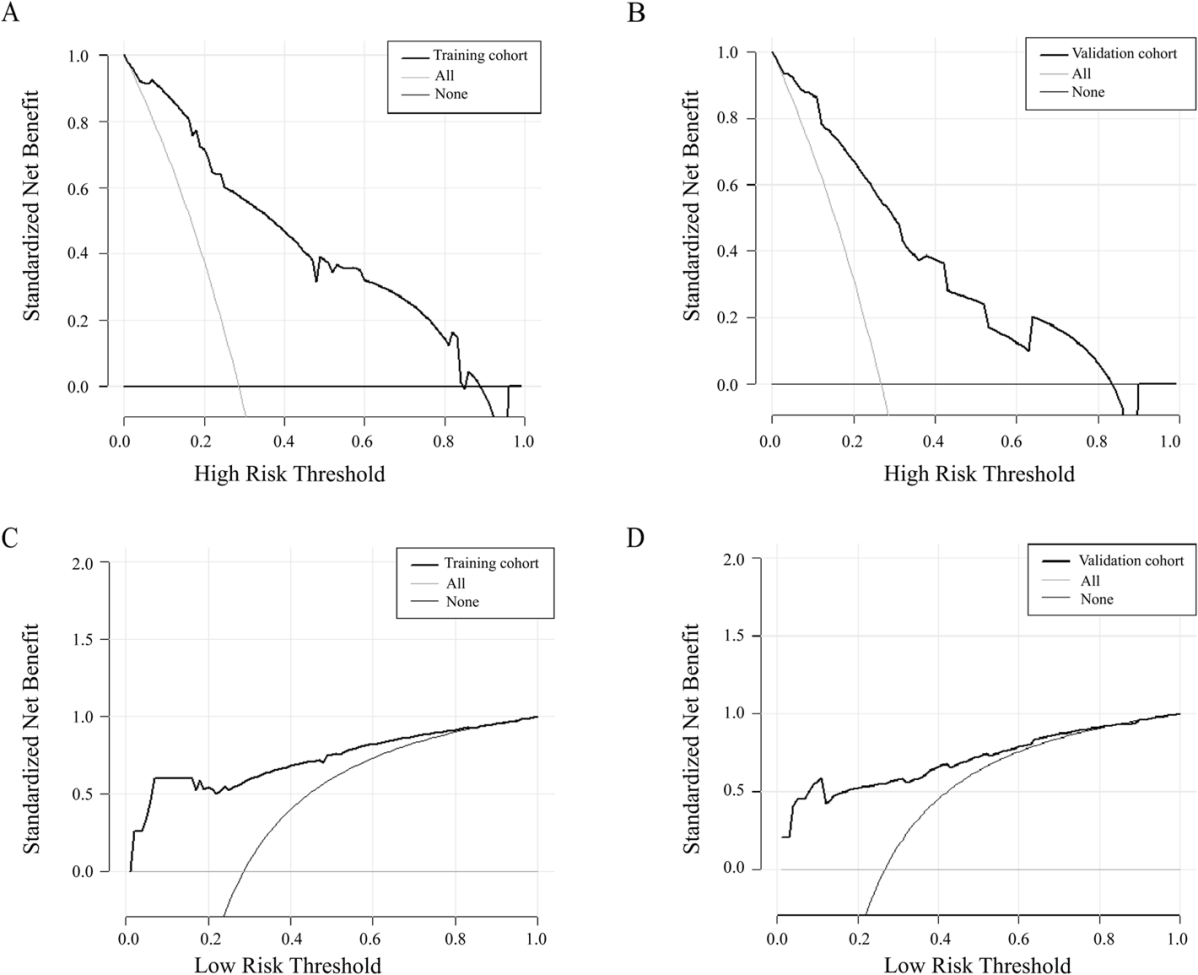
Potential clinical impact of the predictive nomogram for the proposed intervention. The X-axis indicates that the threshold probability refers to the relative harm and benefit of the proposed intervention; the Y-axis indicates that the standardized net benefit. **a**. Decision curve analysis for the training cohort. **b**. Decision curve analysis for the validation cohort. **c**. Opt-out decision curve analysis for the training cohort. **d**. Opt-out decision curve analysis for the validation cohort

DCA for the predictive nomogram model in the training and validation cohorts is shown in Fig. 4a and b, respectively. Based on the curves, the predictive nomogram actually benefits for treating high-risk patients, as indicated by the solid black line above the “treatment for all” line (which was approximately > 0.15 of threshold probability for both cohorts). Opt-out DCA for the predictive nomogram in the training and validation cohorts is shown in Fig. 4c and d, respectively. The predictive nomogram showed a better performance for opting low-risk patients out of treatment, as indicated by the solid black line above the “treatment for none” line (which was approximately < 0.8 and <  0.7 of threshold probability in the training and validation cohorts, respectively).

## Discussion

In the whole cohort during the study period, the infections were mostly caused by *C. albicans*, followed by the *C. glabrata* complex, *C. tropicalis*, *C. parapsilosis* complex, *C. lusitaniae* and others. The crude 30-day mortality was 28.0%, which is comparable to other studies (23.1–35%) [13–15]. Risk factors for death from invasive candidiasis have been studied in recent decades and include older age, hematological malignancy, chronic organ dysfunction, and in the ICU at the time of diagnosis [8]. However, it is difficult to use these risk factors to predict prognosis accurately because we little is known about the weights of these factors in the outcome. A nomogram could solve this problem, and it is commonly used to estimate prognosis in medicine [16]. The user-friendly digital interfaces provide rapid computation for transforming complex clinical parameters into scores, making it easy to understand accurate prognoses and aiding in clinical decision making [17]. In the current study, we established a predictive nomogram (predictor variables were bloodstream infection, ICU admitted > 3 days, no prior surgery, metastasis and no source control) for the prognosis estimation of invasive candidiasis among cancer patients.

ROC analysis is traditionally used to evaluate the performance of a model [18]. Based on the values of AUC, the predictive model constructed in this study has a good ability for prognosis prediction (the AUC was 0.895 in the training cohort and 0.862 in the validation cohort). However, an AUC alone is not sufficient to show that a model would improve decision-making. Vickers and Elkin introduced DCA to estimate the clinical utility of models firstly, and this approach has been widely used for evaluating predictive models and diagnostic tests in recent years [19]. Indeed, DCA can assess the net benefit of nomogram-assisted decisions at different threshold probabilities compared to the net benefit of decisions made with the assumption that the outcome of interest occurs for either all patients or no patient. Another common formulation of DCA is based on an opt-out framework (referred to as opt-out DCA), which displays the population net benefit of the risk model in comparison to the reference policy of treating no patients and opting low-risk patients out of treatment [20–22]. In the training and validation cohorts of the current study, DCA showed that the predictive nomogram performs better than “treatment for all”; opt-out DCA indicated that the model benefits a decision to not undergo some intervention when treat none is the standard. Overall, the current predictive nomogram exhibited good performance regarding outcome prediction and can be applied for some opt-out strategies for invasive candidiasis among cancer patients.

The predictive nomogram constructed in the present study was based on a cancer population, which has been found to be much more susceptible to invasive candidiasis and shows high mortality. There were five predictors in this model, as follows: bloodstream infection, ICU admitted > 3 days, no prior surgery, metastasis and no source control. ICU admission as a predictor for death was consistent with other studies [7, 23, 24]. Previous articles reported that the crude mortality of ICU patients with invasive candidiasis reaches 42.8–58.6% [1, 25]. Source control refers to all physical actions taken to control a focus of infection, including reducing the burden or growth conditions favorable for microorganisms [26]. Previous studies on abdominal candidiasis found that source control is a crucial determinant of survival, and the absence of adequate abdominal source control has been associated with mortality [26, 27]. In the current study, we also confirmed that no source control is a predictor for mortality due to invasive candidiasis.

Bloodstream infection as a predictor of 30-day death from invasive candidiasis has rarely been reported. Invasive candidiasis is commonly divided into bloodstream and deep-seated infections, such as intra-abdominal and intrathoracic abscesses, organ infections or osteomyelitis [5]. Compared to bloodstream infection (removing the intravenous catheter is the common method for source control, but it can only provide transient fungal burden reduction, and the source of infection is difficult to define most of the time), deep-seated infections in the current study occurred at the abdominal, thoracic and pelvic cavities. Source control, such as drainage (could continuously reduce the fungal burden), is easy to perform, which is important for a successful outcome [28]. This might partially explain why deep-seated infections had low mortality and bloodstream infections were associated with a poor prognosis in this study. A surveillance investigation also reported a low mortality of invasive candidiasis (30-day mortality rate of 12.3%), and the researchers considered it to be associated with a low ratio of candidemia and ICU stay [23].

As one of the predictors, metastasis has always been found accompanied by immunosuppression, which is associated with grim outcomes of infections due to *Candidia spp.* [29, 30]. On the other hand, previous study proved that *Candida spp.* could also accelerate the cancerous processes [31]. Thus, we speculated that the poor outcomes depend on the interaction of cancer metastasis and invasive candidiasis, which need to be further studied.

Interestingly, we found that patients who did not undergo surgery had a poor prognosis in invasive candidiasis. Previous studies also found that surgery was associated with a higher probability of survival [11, 32, 33]. To our knowledge, surgery is usually performed on patients without severe underlying diseases, especially among cancer patients. This might be the reason for the patients who were treated with surgery had favorable outcomes. However, one study reported that surgery was associated with high mortality of invasive candidiasis [24]. The discrepant findings might be associated with the types of surgery in the study population, as Orsetti E et al. pointed out that the mortality of candidemia varies among surgery types [34].

In general, hematologic malignancy and fluconazole prophylaxis were not associated with the outcome of invasive candidiasis in this study, which was inconsistent with some investigations for hematologic malignancy that related a poor outcome and fluconazole prophylaxis to a favorable outcome [11, 35]. This might be partially explained by the limited number of cases (13 cases of hematologic malignancy and 20 cases of fluconazole prophylaxis) in our study, and the association needs further investigation. Protection against poor outcomes was also not related to timely antifungal treatment in the study, and we focused on 30-day crude mortality rather than mortality attributed to invasive candidiasis among cancer patients might be explained this. Death might mainly be due to cancer, and invasive candidiasis merely drives progression, which may have reduced the rate of successful therapy of timely antifungal treatment in the study population. A previous study also reported that antifungal treatments significantly reduced mortality for the first 7 days (most of the morality attributable to invasive candidiasis would be expected to occur within approximately 7 days) but showed no significant differences in 30-day mortality [36].

Among the whole population of this study, *C. albicans* was the predominant species, consistent with most recent reports [37, 38]. However, the *C. glabrata* complex ranked second, which was not highly consistent with other studies, with differences in population, region and study design; for example, some articles report that *C. tropicalis* is the dominant species in infections caused by non-*albicans Candida* [7]. Moreover, *Candida* species were not linked to 30-day crude mortality in the present study. Nonetheless, other studies found some relationships between *Candida* species and mortality. Viscoli C et al. observed that the *C. glabrata* complex was associated with the highest mortality rate compared with other *Candida* species [39], and Tedeschi S et al. reported that infection due to *C. tropicalis* was an independent risk factor for in-hospital mortality [40]. Hirano R et al. found that *C. albicans* was associated with a high mortality rate [33]. Khatib R et al. showed that the 30-day mortality rate was not different between *C. albicans* and the *C. glabrata* complex [41]. Combining these findings, we consider that the specificity of *Candida* species-related mortality is associated with the study cohort rather than the species itself, i.e., the initial azole monotherapy was associated with worse outcome for cancer patient infected by fluconazole-susceptible dose-dependent *C. glabrata* complex [42].

## Conclusions

In conclusion, the crude 30-day mortality of the whole cohort was 28.0%, and variables including bloodstream infection, ICU admitted > 3 days, no prior surgery, metastasis and no source control were predictors for mortality. Based on these predictors, we established a predictive nomogram that has good performance in prognosis prediction as well as clinical treatment strategies for invasive candidiasis among cancer patients. We believe that the predictive nomogram will benefit outcome improvement. However, there are still several limitations in this study. This was a single-center investigation that did not use an external population to validate the predictive nomogram. In the future, a multicenter study with external validation should be performed, which may provide more relevant epidemiological information.

## Data Availability

The datasets used and analyzed during the current study are available from the corresponding author on reasonable request.

## Abbreviations

AIC: Akaike’s information criterion
AUC: The area under the receiver operating characteristic curve
CVC: Central venous catheter
CI: Confidence interval
C-index: Concordance index
DCA: Decision curve analysis
ICU: Intensive care unit
LOS: Length of hospital stay
ROC: Receiver operating characteristic
TPN: Total parenteral nutrition
